# Supplementary material for: Patterns of Intron Gain and Loss in Fungi
Source: PLoS Biol. 2004 Nov 30;2(12):e422. doi: 10.1371/journal.pbio.0020422 (PMC532390; doi:10.1371/journal.pbio.0020422)
Supplement: Table S1 — Also available at http://genes.mit.edu/NielsenEtAl/. (4.3 MB ZIP). [file pbio.0020422.st001.zip › NielsenEtAl/html/73.html]

AN5563.1.NCU04923.1.MG00097.1.FG07908.1


```
 CLUSTAL W (1.82) Multiple Sequence Alignments - Introns Inserted


Sequence 1: MG00097.1	323 aa
Sequence 2: FG07908.1	256 aa
Sequence 3: AN5563.1	325 aa
Sequence 4: NCU04923.1	331 aa
Alignment Length: 332 aa
Number Identitical Residues: 143 aa
Alignment Score (without introns) 8007


MG00097.1 	-MPATLANKTFKLNNGVEIPAVGFGTFAAEGQPGQTYAATKAALEAGYRHLDCAWFYQNE
NCU04923.1	MTGFTAANTTYTLNNGVRIPAVGFGTFANEGAKGETYAAVKKALEVGYRHLDCAWFYQNE
FG07908.1 	------------------MPGVGFGTFANEGAKGETYKAVIAALKTGYRHLDCAWFYLNE
AN5563.1  	----MSSGKTFKLSNGVTIPAVGFGTFASEGASGETYRAVKKALEVGYRHLDCAWFYQNE
          	      :..: . ...  :*.******* **  *:** *.  **:.*********** **

MG00097.1 	DEIGNAIADFLKENPSVKREDLFICTKVWNHMHAPEDVKWSLDNSLKALRLDYVDLFLVH
NCU04923.1	DEVGQALAEFLENHKDVKREDIFICTKVWNHLHEPEDVKWSLQNSLDKLKVDYVDLFLIH
FG07908.1 	GEVGQAVRDFLAEDNGVKREDLFICTKVWNHLHEPEEVKWSFENSLKNFGLDYIDLFLVH
AN5563.1  	DEVGDAVRDFLKENPSVKREDIFICTKVWNHLHRPEDVRWSIEDSLKKLKTDYVDLFLIH
          	.*:*:*: :** :. .*****:*********:* **:*:**:::**. :  **:****:*

MG00097.1 	WPIAAERTEDRQ-VKLGPDGK~YVINHELTENPEPTWRAMEELYEAKKARAIGVSNWTID
NCU04923.1	WPIAAEKDEATNMPKIGPDGK~YIIKKELTENPEPTWRAMEDLVDAGKTRSIGVSNWTIP
FG07908.1 	WPIAAEKGDDYQ-PKIGPDGK~YVIKKDLTENPEPTWRAMEEIYASGKARAIGVSNWTID
AN5563.1  	WPIAAEK-ESQDKPKIGPDGK0YVILKDLTEDPKPTWQAMEKLYEDKLARSIGVSNWTIE
          	******: :  :  *:***** *:* ::***:*:***:***.:     :*:******** 

MG00097.1 	GLKKLFAVAKVKPAVNQIEIHPYLPNEELVRFCLDNDVLPSAYSPLGSQDQVPTTGERVR
NCU04923.1	GLQKLLKFARIKPTVNQIEIHPFLPNTELVEFCFKNQIIPTAYSPLGSQNQVPSTGERVR
FG07908.1 	GLKQLMAFAKVKPAVNQIEIHPFLPNEDLVKFCLENDILPAAYSPLGSQNQVPTTGETVR
AN5563.1  	GLEKLLKYAKVKPHVNQIEIHPFLPNEELIQYCWKNDILPEAYSPLGSQNQVPTTGERVS
          	**::*:  *::** ********:*** :*:.:* .*:::* ********:***:*** * 

MG00097.1 	DDPGLNAVANRSNMTLAQALLGWGVKRGYV~VLPKSSTPSRIKSNIEVPDLSEADYQDLW
NCU04923.1	DDPTLKAVAERSGHNLAQVLLAWGLRRGYV~VLPKSSTPSRIESNFQIPVLRDEDFKAIQ
FG07908.1 	SNKTLNEVADRSGNTLAQVLIAWGLRRGYA0PVRRGL-----------------------
AN5563.1  	ENKTLNEIAQKGGNTLAQVLIAWGLRRGYV~VLPKSSNPARIESNFKTIELSDEDYEAVN
          	.:  *: :*::.. .***.*:.**::***.  : :. ..:  .:. .     . . .   

MG00097.1 	KVANGRKPTRFVDMKDTFGYDLWKESQ-------
NCU04923.1	EVAKGR-HCRFVNMKDTFGYDVWPEESDGQLKQE
FG07908.1 	----------------------------------
AN5563.1  	AVAKGR-HFRFVNMKDTFGYDVWPEETAKNISA-
          	  :..       . ..: . .   ..   . .
```
